# Fatty Acid-Stimulated Insulin Secretion vs. Lipotoxicity

**DOI:** 10.3390/molecules23061483

**Published:** 2018-06-19

**Authors:** Petr Ježek, Martin Jabůrek, Blanka Holendová, Lydie Plecitá-Hlavatá

**Affiliations:** Institute of Physiology of the Czech Academy of Sciences, 14220 Prague, Czech Republic; martin.jaburek@fgu.cas.cz (M.J.); blanka.holendova@fgu.cas.cz (B.H.); lydie.plecita@fgu.cas.cz (L.P.-H.)

**Keywords:** fatty acids, fatty acid-stimulated insulin secretion, GPR40, pancreatic β-cells, oxidative stress, lipotoxicity, type 2 diabetes, low-grade inflammation

## Abstract

Fatty acid (FA)-stimulated insulin secretion (FASIS) is reviewed here in contrast to type 2 diabetes etiology, resulting from FA overload, oxidative stress, intermediate hyperinsulinemia, and inflammation, all converging into insulin resistance. Focusing on pancreatic islet β-cells, we compare the physiological FA roles with the pathological ones. Considering FAs not as mere amplifiers of glucose-stimulated insulin secretion (GSIS), but as parallel insulin granule exocytosis inductors, partly independent of the K_ATP_ channel closure, we describe the FA initiating roles in the prediabetic state that is induced by retardations in the glycerol-3-phosphate (glucose)-promoted glycerol/FA cycle and by the impaired GPR40/FFA1 (free FA1) receptor pathway, specifically in its amplification by the redox-activated mitochondrial phospholipase, iPLA2γ. Also, excessive dietary FAs stimulate intestine enterocyte incretin secretion, further elevating GSIS, even at low glucose levels, thus contributing to diabetic hyperinsulinemia. With overnutrition and obesity, the FA overload causes impaired GSIS by metabolic dysbalance, paralleled by oxidative and metabolic stress, endoplasmic reticulum stress and numerous pro-apoptotic signaling, all leading to decreased β-cell survival. Lipotoxicity is exerted by saturated FAs, whereas ω-3 polyunsaturated FAs frequently exert antilipotoxic effects. FA-facilitated inflammation upon the recruitment of excess M1 macrophages into islets (over resolving M2 type), amplified by cytokine and chemokine secretion by β-cells, leads to an inevitable failure of pancreatic β-cells.

## 1. Introduction

The Janus face role of fatty acids (FAs) in relation to insulin secretion and the development of pre-diabetic and diabetic states of type 2 diabetes are discussed in this review. We summarize the simple fact that, on the one hand, the long chain C16–C18 FAs (LCFAs) are the most efficient stimulants of the insulin release in pancreatic β-cells [1,2], whereas, on the other hand, elevated LCFA concentrations in obesity via elevated oxidative stress and low-grade inflammation result in impaired insulin secretion and may lead to the disease progression of fully developed type 2 diabetes [3]. The direct effect of various FAs on β-cell function is complex and pleiotropic. It depends on the chemical nature, concentration, exposure time, and interaction with other nutrients [4]. These aspects are discussed in detail below.

Type 2 diabetes is a very complex disease [5] involving components of the impaired β-cell and pancreatic islet biogenesis and homeostasis; impaired hormonal (endocrine, paracrine, and autocrine) effects on β-cells, but also other islet cell types; components of the chronic low-grade inflammation [6,7,8], typically in white adipose tissue (WAT), but also involving pancreatic islet inflammation; plus the manifested insulin resistance of peripheral tissues [9]. As a result of this complexity, we will focus in this review only on the components of the oxidative and metabolic stresses impacting pancreatic β-cells and low-grade inflammation in pancreatic islets. The molecules in focus will be long chain fatty acids.

### 1.1. Canonical Mechanism of Insulin Secretion

#### 1.1.1. Glucose-Stimulated Insulin Secretion (GSIS)

The glucose sensor of pancreatic β-cells is substantiated, namely, by the elevated ATP as a result of the increased oxidative phosphorylation (OXPHOS) upon the glucose intake from the basal levels of 3.9 mM in humans, and 5.5 mM in mice [1,10,11]. Unlike in other cell types, the constant glucose intake, equilibrating the capillary blood glucose levels with the β-cell cytosolic levels, is ensured by the glucose transporter GLUT2 in rodents and possibly by GLUT1 in humans. The increasing ATP/ADP ratio affects the plasma membrane ATP-sensitive K^+^ channel (K_ATP_), which closes and initiates concerted events of several other channels, leading to plasma membrane depolarization [12]. This activates voltage-gated l-type Ca^2+^ channels (Ca_L_), causing Ca^2+^ entry and resulting in Ca^2+^-dependent exocytosis of the insulin-containing secretory granules. The latter may be augmented (or initiated), for example, at the glucagon-like peptide 1 (GLP-1) stimulation via the GLP-1 receptor, Gs protein, and the cyclic AMP- (cAMP)-dependent activation by the protein kinase A (PKA) and the exchange protein directly activated by cAMP 2 (EPAC2) [13]. Alternatively, the glucose-dependent insulinotropic peptide (GIP) acts in a similar way [14]. Similarly, the acetylcholine via muscarinic M3 receptors, ATP via purinoreceptors, and LCFAs or other metabolites via metabotropic receptors (GPRs, see below) act by stimulation of the Gq proteins and the Ca^2+^ release independent of K_ATP_ [11]. Also, the glycerol/fatty acid cycle that is relevant for fatty acid insulin secretion, acts independently of K_ATP_ [1]. Notably, an inhibitory mechanism for insulin release exists, acting via the inhibitory Gi proteins upon the activation of α2-adrenergic receptors or somatostatin receptors [11].

Since the glycolytic enzyme hexokinase IV (glucokinase) in β-cells is not inhibited by the glucose-6-phosphate [15], the lack of such feedback of the inhibition of glycolysis directly connects glycolysis to pyruvate. Consequently, the β-cell respiration and OXPHOS rates are directly related to the availability of glucose. Note also that most of the other cell types demand respiration and metabolism rates according to their needs and not according to the substrate availability, as do the β-cells. Particularly glucokinase enables that glycolysis amplifies a triggering pathway of glucose-stimulated insulin secretion, through the action of the increased cytosolic Ca^2+^ [15].

The recently discovered aspect has explained the adjustment of the range of glucose concentrations for the glucose sensor [16]. Surprisingly, this is ensured by the inhibitory factor protein IF-1 of the mitochondrial ATP-synthase. The ablation of IF-1 allowed a ‘premature switching on’ of the sensor (i.e., elevation of respiration and OXPHOS), just beginning from the zero glucose concentrations. By a weak inhibition of the ATP-synthase (and hence, OXPHOS), IF-1 thus ensures that the mitochondrial respiration and the resulting ATP synthesis starts to increase sharply at ~3 mM glucose levels and saturates at ~12 to 15 mM glucose. In these saturating glucose concentrations, the maximum OXPHOS takes places with the established maximum respiration and maximum mitochondrial inner membrane potential. At the fasting states with ~5.5 mM glucose levels, β-cell respiration is relatively low, as well as the intensity of the ATP synthesis [17].

Also, lipidomics have elucidated details of insulin granule exocytosis. It has been demonstrated that the negatively charged phospholipids (e.g., phosphatidylserine) promote the interaction of the positively charged regions in the membrane soluble N-ethylmaleimide-sensitive factor attachment protein receptor (SNARE) proteins of the insulin secretory vesicle, with the positively charged regions in the SNARE proteins in the plasma membrane [18]. In this way, insulin exocytosis is facilitated by the phosphatidylserine enrichment of the secretory granules.

#### 1.1.2. Impaired GSIS in Type 2 Diabetes

Out of the scope of this review, the etiology of type 2 diabetes is not fully understood [19]. For decades, advances in research have preferred either a predominant component of insulin resistance [20], occurring as a consequence of low-grade inflammation, causing dysfunctional insulin receptor signaling; or, on the other hand, the impaired biogenesis and dysfunction of pancreatic β-cells was emphasized, including, among numerous other factors, the key role of damaged mitochondria [21,22,23] and pancreatic islet inflammation (see Section 3.2). In reality, both the peripheral insulin resistance and dysfunction of pancreatic β-cells as a result of various factors, including β-cell de-differentiation [24], act in concert. Disease development proceeds most probably in an intermittent manner in a ‘spiral of events’, when any small disturbance of, for example, β-cell function/biogenesis, can be projected as initiating insulin resistance, which strikes back and promotes a further turn of β-cell dysfunction and viability deficiency or de-differentiation. Numerous turns of such a vicious spiral lead, via pre-diabetic states, to fully developed type 2 diabetes. The original event might be low-grade inflammation starting in WAT, as is the case with metabolic syndrome. However, since non-diabetic (‘healthy’) obese patients are also frequent, it must initiate as well as defend against diabetes in other ways.

## 2. Physiological Involvement of LCFAs in Insulin Secretion

### 2.1. FA-Stimulated Insulin Secretion (FASIS)

#### 2.1.1. Dietary vs. Cleaved FAs as Relevant Secretagogues for Insulin Secretion

The transformed dietary fat lipids in the form of triglyceride-rich chylomicrons are cleaved locally in the pancreatic islet capillaries by lipoprotein lipase to 2-monoacylglycerol (2MAG) and LCFAs [25,26,27,28]. The 2MAG stimulates the G-protein coupled receptor (GPR), GPR119, residing on the β-cells [29]. The GPR119, via stimulating the stimulatory Gs protein, augments insulin secretion by cAMP-dependent pathways. LCFAs were supposed to directly activate the GPR40/FFA1 (free FA1) receptor, (i.e., another receptor of β-cells stimulating insulin granule exocytosis via the Gq protein), but also via the Gs and arrestin pathways [30,31,32,33,34]. Activation of GPR40 is further relayed to the signal-regulated kinase 1 and 2 (ERK1/2) [35]. The GPR40 ablation or point mutation in mice led to the impaired insulin secretion that was stimulated by FAs, while only mice with the point mutation had normal GSIS [36]. The GPR40 downstream signaling only slightly increases respiration [37]. Secretory phospholipases A2 might also contribute to FASIS, which is similar to phospholipase C [38]. LCFAs are typically imported into β-cells by the CD36 FA transporter. Acetylation/deacetylation may regulate the function of CD36 [39], hence, the NAD^+^-activated sirtuins, as lysine deacetylases, should promote the FA intake into β-cells.

In model β-cells and with isolated pancreatic islets (ex vivo, Jabůrek et al., unpublished, and preliminary data reported in [40]), a novel phenomenon of signal amplification for GPR40 has been reported [2]. Physiologically relevant concentrations of exogenous palmitic acid were not able or sufficient to directly activate the GPR40 to stimulate insulin secretion, but instead were metabolized by the mitochondrial β-oxidation. The latter is pro-oxidant as it produces superoxide at the E_F_ site of the electron-transferring flavoprotein, Q oxidoreductase system (ETFQOR) [41]. The resulting superoxide is converted by superoxide dismutase MnSOD to H_2_O_2_. Such a redox (direct H_2_O_2_) signaling activates the mitochondrial-specific calcium-independent phospholipase A2, isoform γ (iPLA2γ). The active iPLA2γ is able to cleave free FAs from the mitochondrial inner membrane phospholipids, including cardiolipin, and leaves lysophospholipids. Consistent with the known properties of transmembrane FA transport, the intracellular cleaved FA can equilibrate rapidly within the cellular compartments, and it has been demonstrated that the FAs diffuse in and out of the β-cells within a minute [42]. Thus, at least ex vivo, free FAs can diffuse out of mitochondria to the plasma membrane and stimulate GPR40, and subsequently, insulin secretion [2]. However, this mechanism has yet to be demonstrated to exist in vivo.

**Figure 1:**
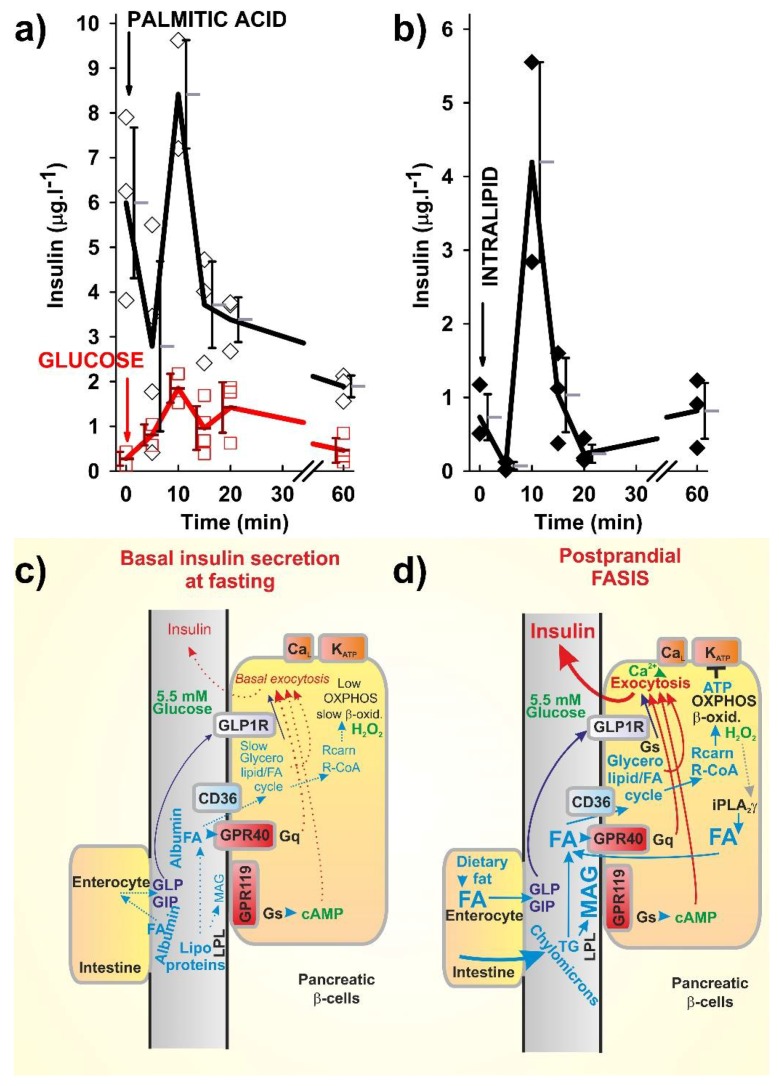
Fatty acid (FA)-stimulated insulin secretion (FASIS) in mice (**a**,**b**) as compared to glucose-stimulated insulin secretion (GSIS), (**a**) and schemes of incretin-mediated basal insulin secretion at fasting (**c**) and FASIS at basal glucose levels (**d**), with a theoretical fat only meal. (**a**,**b**) Time course of insulin release into the blood from the eye plexus blood vessel in C57Bl6J mice, fasted 6 h prior to an intraperitoneal (i.p.) injection (arrows) of 0.1 mg palmitic acid (**a**, black) or 1.5 mg intralipid (**b**) per 1 g of mice body weight, is plotted as the obtained data or the averages with standard deviations. Alternatively, glucose 1 mg per g body weight was i.p. injected (**a**, red). Insulin was detected from the blood serum by an insulin kit (Mercodia, Uppsala, Sweden). Approved by the Animal Care and Use Committee (Inst. Molecular Genetics, ASCR), in accordance with the European Union Directive 2010/63/EU. (**c**,**d**) Insulin release prior to and after a fatty meal not containing saccharides—predicted mechanisms of negligible fasting fatty acid-stimulated insulin secretion (FASIS) vs. significant postraprandial FASIS: (**a**) FASIS due to the existence of FAs in plasma during fasting (‘fasting basal FASIS’) is a part of the basal insulin secretion. Such a very low contribution to the insulin released during the fasting state may originate from the FA stimulation of the intenstinal incretin release with subsequent GLP1R- and GIPR-mediated insulin release in β-cells. (**b**) Postprandial FASIS can also be considered as consisting of the intenstinal and β-cell components—a much higher induction of intenstinal incretin release is now in effect. Solely β-cell-dependent FASIS is now initiated by the lipoprotein lipase cleavage of triglycerides from chylomicrons to long chain C16–C18 FAs (LCFAs) and 2MAG, both stimulating their own receptors (GPR40 and GPR119, respectively), which further stimulates insulin granule exocytosis in K_ATP_-independent ways, as well as evoking metabolic stimulation of the insulin release, after the FA transport into β-cells by the CD36 transporter. The metabolic stimulation proceeds either via the glycerol/FA cycle (part of which stimulates the insulin release in a K_ATP_-independent way via the exocytosis-promoting protein Munc13-1, and part via the corresponding fractional increase in ATP) or after β-oxidation and, subsequently, increased the OXPHOS (hence, ATP) by the K_ATP_-dependent way. Dotted arrows represent low or non-existing signaling or fluxes.

If this amplification exists in vivo, it would coordinate the balance between the two branches of FA-stimulated insulin secretion (FASIS), namely: the ATP-dependent branch, which is automatically K_ATP_-dependent; and the glycerolipid/FA cycle and GPR40-dependent branch, which is predominantly K_ATP_-independent. Thus, the FA metabolism leads to (i) the elevated ATP, produced because of the increased mitochondrial oxidative phosphorylation (OXPHOS) during the FA β-oxidation; and the (ii) stimulated GPR40 by the FAs that returned to the plasma membrane from each possible point of the metabolic pathways (with free FAs as reaction products), and by ‘amplifying’ the FFAs cleaved off phospholipids, reportedly in mitochondria and possibly also in peroxisomes. If one considers also the contribution of either chylomicron-derived 2MAG or 2MAG, originating from the glycerolipid/FA cycle, all of these stimuli belong to the components of the in vivo net FASIS (Figure 1).

However, the FA metabolism in pancreatic β-cells may be specific in preferring the activation of the glycerolipid/FA cycle upon the intake of glucose plus FA. The glycerolipid/FA cycle releases 2MAG, which, via the exocytosis-promoting protein Munc13-1, also stimulates the insulin granule exocytosis [1]. Mitochondrial β-oxidation can be considered apparently as downstream of the glycerolipid/FA, since the human islets perfused at zero glucose do not increase respiration upon the LCFA addition, but do release insulin as a response to the LCFAs [43]. Both respiration and insulin release are increased upon the LCFA addition to human islets in the presence of 5.5 mM glucose. Recently, a novel enzyme, glycerol-3-phosphatase, has been discovered that produces glycerol and thus regulates glycolysis, cellular redox state, ATP production, and other important branches of metabolism [44]. Dysbalance within the glycerolipid/FA cycle is considered to induce insulin resistance, islet β-cell failure, and type 2 diabetes.

**Figure 2:**
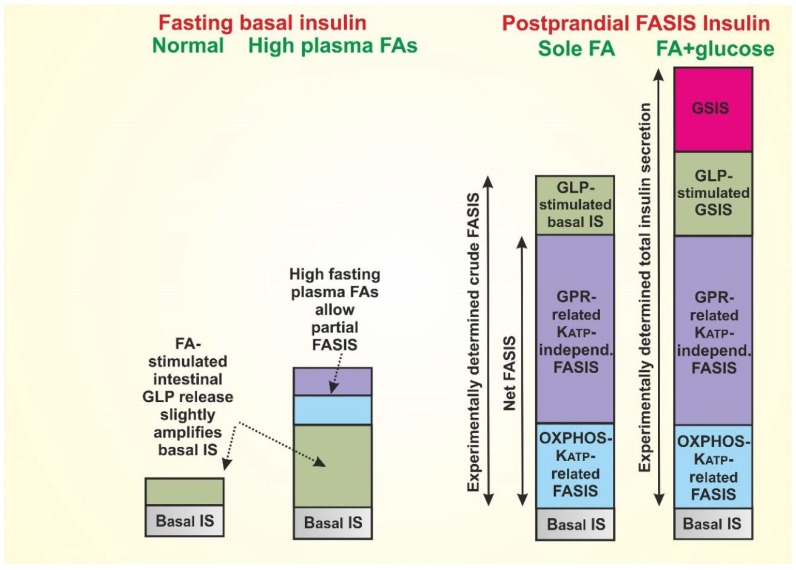
Predicted amount of insulin released by the mechanisms described in Figure 1 (based on References [2,40] and unpublished observations of GLP-1-induced insulin release in fasted mice, by Plecitá-Hlavatá et al.), as follows: **1st column**: Basal, very low, insulin secretion at fasting can be considered partly as originating from the FA stimulation of the intenstinal incretin release with subsequent GLP1R- and GIPR-mediated insulin release in β-cells. We predict that at the lipid overnutrition and metabolic syndrome (**2nd column**), the elevated plasma FAs at fasting will significantly increase this insulin secretion by regular FASIS mechanisms, thus contributing to developed hyperinsulinemia. **3rd column**: Postprandial FASIS in healthy subjects stems from both instenstinal and β-cell components, and is likely to be much higher in comparison with the regular GSIS [2]. Thus, a typical meal with fat and saccharide components (**4th column**) provides a maximum insulin secretion, where the incretin component is elevated and regular GSIS is superimposed onto FASIS. Definitions of ‘net FASIS’, ‘crude FASIS’, and total insulin secretion are shown. Note also that time dependence is not considered, thus the typical 1st phase and 2nd phase of insulin release can be composed by different glucose-dependent, incretin-dependent, and FA-dependent fractions.

In addition, it is also necessary to inspect whether the minimum levels of FAs that are bound to plasma albumin upon ongoing GSIS also activate insulin secretion by such a basal FASIS. Upon glucose intake, FA levels are minimum due to the inhibited lipolysis in WAT, when adipocyte lipases are inhibited by insulin receptor signaling at the expense of the glucose uptake [45]. In contrast, in the fasting state, WAT lipolysis promotes higher ‘fasting levels’ of FAs. Hence, it must be investigated whether there is a FASIS component due to these ‘fasting FA levels’, contributing to the minimum (basal) insulin release. Moreover, it should be determined whether overnutrition and obesity increases this basal insulin release (Figure 2). One should ask whether the FASIS component in it is increased as a result of the increased plasma FAs? Finally, postprandial levels of FAs bound to albumin should be high (though they may increase first and then decline), and it should be recognized that not only the FA intake facilitated by lipoprotein lipase from chylomicrons, but also simple transfer from the FA bound to albumin, is relevant for postprandial FASIS.

#### 2.1.2. Experimental Determination of FASIS

However, even at basal glucose with an experimental dosage of LCFAs (Figure 1a,b), the determined blood insulin release, the obtained total FASIS (i.e., ‘crude FASIS’), contains components of the ‘net FASIS’ (i.e., β-cell specific, incretin-independent FASIS, above the fasting basal FASIS, if it exists), given by both the K_ATP_-dependent and K_ATP_-independent mechanisms, as described above, including the GPR40 pathway (GPR120 [46] and the glycerolipid/FA cycle might also be involved, as well as a component of the incretin-stimulated insulin secretion at basal 5.5 mM glucose) (Plecitá-Hlavatá et al., unpublished). 

We repeat that the latter exists because of the intenstinal stimulation of incretin secretion by FAs. Indeed, the LCFAs cleaved off the dietary fat stimulate the enterocyte GPR120/FFA4 receptor, thus inducing the secretion of incretins GLP-1 [13] and GIP [14], both of which amplify the GSIS in β-cells via their receptors. Bile acids also stimulate the enterocyte incretin secretion via the TGR5 receptor [13]. Consequently, experimentally, the ‘net FASIS’ might be evaluated only at the blocked GLP-1 and GIP receptors (Figure 2).

As considered above, one may ask reciprocally whether the relatively higher blood circulating LCFAs at fasting also stimulate the basal insulin secretion at fasting glucose via the enterocyte GPR120/FFA4 receptor and ‘basal GLP-1’? These basal events would explain the basal insulin secretion, which are given similarly by the GLP-1 (GIP) component, and the GPR40 and glycerolipid/FFA cycle components. Simply, the fasting basal levels of a healthy organism result from these basal equilibria of hormones, metabolites, and cell homeostasis at fasting. In states of the impaired glucose tolerance, this basal fasting blood glucose is already slightly higher than 5.5 mM, whereas with developed type 2 diabetes, significant hyperglycemia exists. Of course, both of these pathologies would elevate the basal GLP-1-related GSIS plus basal FASIS because of ‘fasting FAs’, acting via the GPR40 pathway. Consequently, such a situation can further perpetuate the continuation of pathology development.

Previously, FAs were considered only as amplifiers of GSIS in the absence of pathological states [47,48,49]. Indeed, supraphysiological experiments at zero glucose with human pancreatic islets gave only halved insulin responses on added FAs, but lacked the ability to increase the islet respiration [43,50]. However, with basal (5.5 mM) glucose, FA-induced respiration and FASIS occurred [43], as was similar to mice (Figure 1a,b). All of these data reflect the preferential function of the glycerolipid/FA cycle over the FA β-oxidation in pancreatic β-cells. We prefer a physiological definition, considering a theoretical fat-only meal as inducing FASIS at basal (5.5 mM) glucose. With increasing glucose, the pathways of FASIS and GSIS act in concert. In experiments, FASIS may exceed GSIS [2,40]; however, the proportions of these two mechanisms, in response to various human diets, have to be further investigated.

When we recognize the existence of FASIS, despite the difficulties to quantify it experimentally in vivo [37,43], one may get a deeper insight into the interrelationships between the glucose and lipid metabolism. The net FASIS in model pancreatic β-cells, insulinoma INS-1E cells, provides >3 times more insulin than GSIS [1,2,40]. The similar pattern is valid when comparing the intraperitoneal (i.p.) dosage of glucose vs. LCFA (Figure 1a). Thus, despite the higher blood circulating LCFAs at fasting vs. the lower blood LCFAs due to the insulin-induced FA intake into adipocytes, the dietary intenstinal LCFAs (via GLP-1 and GIP) and LCFAs cleaved locally from chylomicrons at β-cell intersticia, should induce a much higher insulin release, when compared with the fasting (and higher) blood circulating LCFAs (Figure 2).

Let us consider now even higher blood circulating LCFAs in obese states. The basal net FASIS should be higher, and at the elevated food intake the crude FASIS must be even higher (Figure 2). In pre-diabetic states, when insulin release is not hampered, the resulting very intense insulin release enhances FA storage into WAT. This is of course facilitated in the absence of skeletal muscle energy expenditure. As stated above, in this way, higher pathological levels are perpetuated.

### 2.2. Specificity of Distinct Classes of Fatty Acids

#### 2.2.1. Polyunsaturated FAs (PUFAs)

As recognized in numerous studies, the FA acute and chronic effects on pancreatic β-cell function are quite complex. The first clear sorting can be carried out in relation to the chemical nature of FA. In the first rough selection, unsaturated vs. saturated FAs can be considered. However, since polyunsaturated FAs (PUFAs) are precursors for the metabolism of a wide spectrum of pro-inflammatory or even anti-inflammatory compounds, and since PUFAs as components of lipids are prone to lipid peroxidation, they should be considered as specific and more complex entities. PUFAs were reported to be more active in numerous aspects, including FASIS, but certain PUFAs are less pro-inflammatory and exert opposite effects with the regard to the induction of insulin resistance [51]. Typically, ω-3 PUFAs may exert different effects than ω-6 PUFAs. Thus, for example, metabolism of ω-3 PUFA, such as eicosapentaenoic acid (EPA), leads to prostaglandin PGE_3_, while the more common PGE_2_ is an arachidonic acid metabolite (i.e., metabolite of ω-6 PUFA). PGE_2_ has been known to reduce GSIS. However, PGE_3_ is nearly without such a lipotoxic effect. In accordance with this, the enrichment by EPA—which decreases arachidonic acid and its metabolites—has a positive, anti-lipotoxic effect, probably due to PGE_3_ signaling [52]. Also, synthetic FA derivatives are tested as anti-lipotoxic agents or agents improving insulin secretion (e.g., artificial GPR40 ligands) [53].

#### 2.2.2. ω-3 Polyunsaturated FAs

The ω-3 PUFAs are recognized to be specific among FAs. ω-3 PUFAs have been reported to prevent or reverse the high-fat diet-induced WAT inflammation and insulin resistance. ω-3 PUFAs also block cytokine-induced β-cell death. Their supplementation thus prevents β-cell destruction and corrects insulin resistance [51]. Multifaceted effects are provided (e.g., by EPA), namely for the potentiation of GLP-1 secretion in L-enterocytes, up-regulation of the apelin pathway, and down-regulation of other pathways, thus enhancing insulin secretion in β-cells [54]. EPA also suppresses inflammatory responses to adipokines and inhibits peroxisome proliferator-activated receptor α (PPAR α) signaling; likewise, potentiating the insulin-like growth factor-1 secretion and thus counteracting the peripheral insulin resistance. The EPA effects in the organism may be largely ascribed to its anti-inflammatory metabolites resolvins. Also, docosahexaneoic acid (DHA) is metabolized into DHA-derived specialized pro-resolving mediators, DHA epoxides, electrophilic oxo-derivatives of DHA, neuroprostanes, ethanolamines, acylglycerols, docosahexaenoyl amides of amino acids or neurotransmitters, and branched DHA esters of hydroxyFAs [55,56,57]. A description of their roles is out of the scope of this review.

#### 2.2.3. Oxidized FAs—Specific Messengers vs. Pathology Markers

Lipidomics describing the very long chain PUFA downstream products is even more complex when considering their oxidized products, which are formed either by specific enzymes (e.g., by lipoxygenases) or by free-radical-initiated non-enzymatic lipid peroxidation. Thus, for example, an eicosanoid metabolite of the ω-6 PUFA arachidonic acid, 20-hydroxyeicosatetraenoic acid (20-HETE), could also be considered as an anti-inflammatory compound. The 20-HETE is present in the human plasma in nanomolar concentrations [58]. Recently, it has been found that pancreatic β-cells form 20-HETE by cytochrome P450-dependent ω-hydroxylases, and such a formation was enhanced at high levels of glucose. Moreover, the 20-HETE was recognized as a more efficient agonist of the GPR40 receptor than the dietary FAs [59]. 20-HETE thus stimulates FASIS and appears to play an autocrine-like role in β-cells.

## 3. Pathology Related to LCFAs

LCFAs affect different aspects of cell physiology. Consequently, the actions of LCFAs and their metabolites, in an exaggerated or impaired manner, can progress into the pathology [60]. Typically, the excessive LCFA levels inhibit GSIS. From a medical point of view, a simpler sequence of events might substantiate the acute lipotoxicity, whereas a longer lasting and more complex sequence of events substantiates chronic lipotoxicity. Both of the lipotoxic effects may also contain a pro-inflammatory component. Experimentally, LCFAs are stimulants for FASIS, but only up to an optimum, (i.e., a threshold above which the insulin secretion with further increasing FA dose starts to decline). Moreover, LCFAs frequently induce oxidative stress or apoptosis [61,62]. Besides these basic effects, the LCFAs may alter cell signaling and membrane composition. All of these events belong to the components of the chronic lipotoxicity. We note again that when in vivo, the latter is typically connected with inflammation. Nevertheless, a pathology threshold concerning certain parameters should be determined. Surprisingly, a wide clinical study did not find any link between plasma-free FAs and obesity or insulin resistance [63]. Perhaps, an individualized medical approach is required to elucidate all of the included pathological components for a given patient. Recent metabolomics approaches would support this individualized medicine.

### 3.1. Oxidative Stress Related to LCFA Metabolism

#### 3.1.1. Pro-Oxidant Role of Fatty Acids

The mitochondrial and peroxisomal β-oxidation of LCFAs provides the first line source of superoxide (O_2_^●−^; and its conjugated acid—hydroperoxyl radical, HO_2_^●^, pKa 4.9), formed as the most upstream among the reactive oxygen species (ROS) [64,65,66,67]. Typically, the superoxide is converted to H_2_O_2_ by superoxide dismutases (SOD1/ZnCuSOD localized in the cytosol and mitochondrial intracristal space [68,69]; plus by SOD2/MnSOD in the mitochondrial matrix) [64]. H_2_O_2_ may be converted by Fenton reaction with iron to the most reactive species—to the hydroxyl radical, ^●^OH, acting locally. Numerous isoforms of selenium-dependent glutathione peroxidases (GPX) convert H_2_O_2_ to water and also convert free hydroperoxy FAs (FAOOHs) to their corresponding hydroxy acids (FAOH), with glutathione (GSH) as a cofactor. The fourth isoform, GPX4, specifically acts on the hydroperoxy groups of the peroxidized phospholipid side chains and on cholesterol hydroperoxides [70,71,72]. Also, peroxisomal H_2_O_2_ belongs to factors that are significantly contributing to the lipotoxicity in pancreatic β-cells [73].

The hydroperoxyl radical, HO_2_^●^ (i.e., conjugated acid of superoxide) and hydroxyl radical ^●^OH are capable of initiating non-enzymatic lipid peroxidation, a second line of ROS sources, in which FAs play a pro-oxidant role [74]. Indeed, hydroperoxyFAs, hydroxyFAs (converted from hydroperoxyFAs by glutathione peroxidase 4, GPX4), and numerous other derivatives of PUFAs coming from enzymatic lipid peroxidation are cleaved from oxidized lipids by phospholipases A2 [75]. Typically, the shorter lipid peroxidation products (e.g., arachidonic acid metabolites) are pro-inflammatory, while the resolvins coming from the C22 ω-3 PUFAs are anti-inflammatory [76]. One would therefore consider the non-enzymatic lipid peroxidation as pro-inflammatory, whereas the enzymatic lipid peroxidation creates more anti-inflammatory compounds.

Lipooxygenases are key enzymes for enzymatic lipid peroxidation and were also implicated in β-cell dysfunction [77,78,79]. The 15-lipoxygenase-1 (ALOX15), and possibly also 15-lipoxygenase-2 (ALOX15B) and 5-lipoxygenase (ALOX5), but also cyclooxygenase-2 (COX-2), and certain cytochrome P450 monooxygenases, are responsible for the conversion of ω-3 PUFAs into resolvins. Resolvins counteract inflammation. In contrast, 4-hydroxyalkenals, as the end products of lipid peroxidation, contribute to diabetic complications [80].

The cell antioxidant defense mechanisms keep the concentrations/amounts of ROS at the physiological level. Thus, in pancreatic β-cells, both the key mitochondrial ROS detoxifying enzymes, MnSOD and GPX, are essential, not only for balancing redox homeostasis, but also for insulin secretion [81]. The disulfide reductases constitute another type of antioxidant defense, namely thioredoxins (TRX), glutaredoxins (GRX), peroxiredoxins (PRX), and glutamate-cysteine ligase. These enzymes are also capable of relaying (spread) the redox signals to the targets. Thioredoxins represent the disulfide reductases for protein sulfhydryl groups, maintaining proteins in the reduced state [82]. Thioredoxin reductase uses electrons from NADPH and regenerates the oxidized TRX. Similarly, glutaredoxin reductase-2 reduces the H_2_O_2_ or hydroperoxy-FA lipid chains to water or hydroxyFA lipid chains, respectively, at the expense of the conversion of GSH to oxidized glutathione GSSG, which is subsequently regenerated by glutathione reductase [83]. Peroxiredoxins are a family of thiol peroxide reductases using TRX or other thiol-containing proteins to clear H_2_O_2_ or lipid peroxides [84]. The catalytic cysteine sulfhydryl group of the PRXs is selectively oxidized by H_2_O_2_ to either sulfenic acid or disulfide intermediates. At the TRX shortage, PRX is inactivated to PRX-SO2, which can be reversed by sulfiredoxins, at the expense of ATP, yielding peroxiredoxin sulfenic acid PRX–SOH [85]. Peroxiredoxins consist of two major enzyme classes, differing by the mechanism of recycling of the sulfenic acid formed back to a thiol. The 2-Cys PRXs are reduced by thiols such as thioredoxins, thioredoxin-like proteins, or, in certain cases, by glutathione. The 1-Cys class of PRXs is reduced by ascorbic acid or glutathione in the presence of GST-π [86]. PRX3 is localized to mitochondria, where, like other PRXs, it participates in the spread of the redox H_2_O_2_ signaling.

Numerous redox signaling events exist, when certain ROS species, typically H_2_O_2_, are transiently elevated above the basal levels and may spread by redox buffers or mediators towards the targets, which may be located even in the different cell organelles or parts [65]. Exemplar redox signaling has been characterized for hypoxia, when the superoxide generated at the Complex III or the respiratory chain of the mitochondria provides a redox signal, oxidizing the Fe^II^ of the proline hydroxylase domain containing enzymes (PHDs), which leads to HIF1α stabilization and initiation of the hypoxic transcriptome reprogramming [87]. Another redox signal has been recently identified for the norepinephrine initiation of thermogenesis in brown adipocytes, when the redox burst targets the cysteine of the mitochondrial uncoupling protein, UCP1, and thus initiates uncoupling and heat production [88]. Redox signaling implicated in pancreatic β-cell function is yet to be studied in detail.

**Figure 3:**
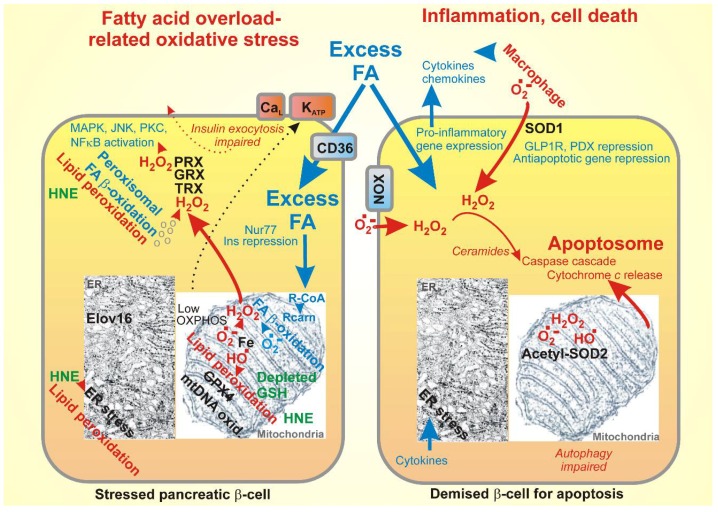
Oxidative stress and cell death originating from FA overload, namely: (**left)** oxidative stress due to FA excess and (**right**) apoptosis resulting from the elevated reactive oxygen species (ROS).

Only the dysbalance leads to so-called oxidative stress, when the ROS production significantly and permanently exceeds the antioxidant mechanisms. A permanent character distinguishes this stress from the repeatable redox signals. For example, the function of succinate dehydrogenase leads to an elevated ROS formation [89]. Oxidative stress possesses not only direct pathological consequences by the more frequent appearance of oxidative products of biological constituents, but also by the induction of the developed demising cellular responses (i.e., programmed cell death, such as apoptosis) (Figure 3).

Oxidative stress leads to oxidative modifications of DNA and to more vulnerable mitochondrial DNA (mtDNA), oxidative modification of lipids (by non-enzymatic lipid peroxidation), and oxidative modification of proteins, such as carbonylation. Despite thephysiological mechanisms clearing these oxidized constituents (repair mechanisms for DNA, GPX, and normal protein turnover), when their accumulation exceeds such clearance, another stress occurs with much more serious consequences. For example, the impaired autophagy due to the palmitate excess [90] or dysregulated specific mitochondrial authophagy, mitophagy, leads to serious constituent stresses [91,92]. Specific types of stresses have been recognized for organelles, such as endoplasmic reticulum (ER) stress, given by the unfolded protein response [93]. LCFAs induce ER stress and apoptosis of β-cells by the Ca^2+^/calpain 2 pathway [94]. The contribution of GPR40 signaling to ER stress was also reported [95].

Cell death mechanisms have developed for situations when the above-described stresses would cause a high burden for the tissue or organism. Apoptosis has been traditionally considered as the major mechanism of regulated cell death [96,97,98], whereas necrosis was characterized as a nonspecific cell death. Recently, another type of cell death has been revealed, termed ferroptosis, occurring only because of specific signals that are provided by lipid hydroperoxides [99,100,101]. It has been proposed that not the apoptosis, but the ferroptosis predominantly causes various neurodegenerative diseases. Concerning β-cells, it is recognized that the disruption of iron homeostasis leads to cell death [102]. It has to be demonstrated whether ferroptosis manifests in certain stresses that impact on β-cells.

ROS also activates stress-sensitive second messengers, such as p38MAPK, JNK [103], and PKC [104]. Oxidative stress also affects transcription factors MAF-A and PDX1, participating in β-cell proliferation and insulin biosynthesis [105,106]. FAs may also induce pro-oxidant NADPH oxidases [107]. Palmitate metabolism also generates ceramides, serving as a signal transduction in ROS-induced apoptosis [108]. Other studies showed that long-chain and saturated acyl-CoA accumulates in the cytoplasm and potentiates the synthesis of ceramides that are implicated in apoptosis and the functional damage of pancreatic β-cells [109]. Ceramides also induce apoptosis through the inactivation of the pro-survival pathways [110]. In general, advances in the study of sphingolipid metabolism have helped to identify the roles that sphingolipids play in pancreatic β-cells. Sphingolipid metabolites, including ceramide, glycosphingolipids, sphingosine 1-phopshate, and gangliosides, modulate the β-cell signaling pathways and are particularly relevant to lipotoxicity [111]. Using mass spectroscopic lipidomics and subcellular fractionation, Boslem et al. have shown that the palmitate pretreatment of MIN6 β-cells promoted ER remodeling of both phospholipids and sphingolipids, but only that the later was causally linked to lipotoxic ER stress [112]. Their results also suggestted that a loss of sphingomyelin in the ER was a key event for initiating β-cell lipotoxicity, which leads to the disruption of the ER lipid rafts, perturbation of protein trafficking, and the initiation of ER stress [112].

#### 3.1.2. Experimental Models of LCFA-Induced Lipotoxicity

In contrast to the fact that LCFAs are the most efficient stimulants of insulin release in pancreatic β-cells, overly elevated LCFAs and their chronic exposure cause the impairment of insulin secretion and, specifically GSIS, as demonstrated both the in vitro and in vivo in animal models and humans [49,113,114,115]. Nevertheless, when choosing a lipotoxicity model, many factors and technical issues have to be considered. Whereas glucose can be conveniently added to cell cultures or injected intravenously, FAs present numerous difficulties for investigators in experimental design and in the interpretation of data [42].

The total serum levels of FAs vary widely (0.2–3 mM), and are transported in the blood mainly in complexes with albumin. In addition, large quantities of FAs are transported from the liver and intestine in the form of triglycerides in chylomicrons and lipoproteins, and are released as free FAs in the capillary endothelium at the sites of utilization [42,116,117]. Albumin serves as a buffer for LCFAs in solution, as long as the albumin concentration exceeds 0.5 μM [118], which is close to the albumin concentration in serum (~600 μM). Thus, when in vivo, the FA production and utilization operates under a dynamic steady-state, which includes a feedback loop between adipose tissue and the pancreas [119]. It has been estimated that about 0.3 mol of FA is transported via the blood plasma from fat tissue to FA-consuming organs every 24 h [120]. On the contrary, in vitro cell models are based predominantly on equilibrium conditions that are determined by the particular molar ratio of the FA and albumin that are used in the system.

The equilibrium binding of LCFAs with albumin has been studied by measuring the equilibrium levels of non-esterified-free FA (FFA), using a fluorescent probe composed of acrylodan-derivatized intestinal FA binding protein (ADIFAB), allowing for the relationship between the concentration of unbound FFA (FFAu) in the water phase and the FFA/albumin molar ratio to be determined [118]. In addition, the unbound free fatty acid levels and FFAuprofiles could be determined in human serum [117,121]. Thus, in humans, the FFAu is typically <10^−5^ of the total FFA [117] and determined that the FFAu values in human serum from healthy donors yielded a mean value of 7.5 nM [117]. To add to the complexity of the experimental parameters that were to be considered in the in vitro or ex vivo systems, the partitioning of free FAs between albumin, aqueous phase, and membranes was also critical [42]. In addition to the issues listed above, recent research has highlighted other technical matters to be considered in the FFA/albumin cell culture lipotoxicity models, as well as the solutions that have been adopted for these problems [122].

Seeking a threshold, experiments using model β-cells or isolated pancreatic islets in the culture allow us to determine the experimental doses of LCFA leading to the stimulation of GSIS and the doses above which the GSIS starts to decline. Thus, by quantification of GSIS at an increasing LCFA dose, one may determine an experimental threshold of the LCFA concentration, leading to an experimental acute lipotoxicity. However, as discussed above, such experiments involve ongoing FASIS simultaneously with GSIS and their interrelationships, together with near equilibrium conditions, that may not be easily translated to in vivo situations.

Nevertheless, the studies using insulinoma INS-1E cells determined the GPR40-related FASIS as accounting for about two-thirds of the insulin release in the absence of glucose, as induced by the total 15 μM palmitic acid, which, in the albumin-containing medium, represented FFA/albumin = 1 and about 1.3 nM of free palmitic acid [2]. Glucose (25 mM), present together with 1.3 nM of free palmitic acid, only slightly elevated both the rate and extent of the nsulin release. However, the net GSIS portion was inhibited by 75% when the FFA/albumin > 6.5, thus exceeding 200 nM of free palmitic acid. The question arises, whether such high free LCFAs can exist locally in any pathological conditions within the pancreatic islet intersticia between the pancreatic β-cells.

Thus, the experimental acute lipotoxicity stems from the elevated LCFA concentrations acting on or within the β-cell that exceed the critical threshold. Another type of experiment is based on a constant FA/albumin and a prolonged (48 h) treatment of the model β-cell lines together with toxicity analyses, based on increased H_2_O_2_ generation and/or selected markers of apoptosis. The toxicity profile of FAs was analyzed using rat as well as human cell lines, and isolated rat and human islets [49,113,123,124]. The choice of cell line is also important, as the very differentiated human β-cell line EndoC-βH1 mimics the in vivo situation, unlike RINm5F and INS1 cells, where unsaturated FAs are poorly metabolized and thus the oleate can antagonize palmitate toxicicty [123].

When using the human EndoC-βH1 β-cell line model, the toxicity was defined as apoptosis initiation (caspase-3, annexin V staining), and only LCFAs (500 μM) with chain lengths >14, which generated H_2_O_2_ in the peroxisomal β-oxidation, were found to be toxic [123]. Unfortunately, those studies refer only to thr total concentrations of FA, which does not allow for a straightforward comparison between different experimental conditions. Nevertheless those studies employed typical standard conditions that could be compared in vitro, that is, a medium supplemented with 2% bovine serum albumin (BSA), corresponding to 0.3 mM BSA in the experiment. Hence, the ratio of 0.5 mM LCFA to 0.3 mM BSA was 1.66. In healthy individuals, under normal physiologic conditions, the value of FA/albumin is ≤1 [117], and the values of FA/albumin ≥1.5 are comparable, for example, with the plasma FA concentrations determined over a postprandial period during a response to a mixed meal with a high fat content [125], or to fasting plasma FA levels that are determined in obese subjects [117,118,126]. Therefore, future experiments mimicking as close as possible in vivo conditions will represent a promising perspective.

In addition to the in vitro studies of FA-induced β-cell dysfunction, the in vivo high fat models are very important for understanding the FA-induced toxicity, as the in vitro conditions cannot mimic the complexity of the physiological FAs’ turnover. There are several in vivo studies, including few data from human studies, which utilize intravenous fat infusion or fat emulsion ingestion to determine the effect of saturated, monosaturated, and polyunsaturated FAs (PUFAs) on β-cell function (reviewed in [114]). It has been determined that, in humans, following the oral ingestion of the three fat emulsions over 24 h, plasma FAs were elevated by ~2-fold over the basal level and only PUFA ingestion resulted in an absolute decrease in GSIS [127].

#### 3.1.3. LCFA Metabolism in β-Cells May Cause Lipotoxicity

The precise mechanism through which FAs generate functional damage in β-cells needs to be clarified. Pancreatic β-cells are vulnerable to oxidative stress, since their defense antioxidant mechanisms are set to low capacities [128]. We may speculate that this has developed to promote redox signaling much more easily. Nevertheless, the low-capacity antioxidant system can be easily overcome, and severe oxidative stress may at least impair physiological functions of β-cells, such as glucose sensing and insulin secretion. A notorious example is the inhibition of insulin release by excessive LCFAs. The molecular mechanism is not yet known in detail, and the exact thresholds for the inhibitory role of LCFAs in contrast to their stimulating role in FASIS are yet to be determined. Despite the important consequences of oxidative stress for β-cells, administration of cytosolic antioxidant *N*-Acetylcysteine does not improve glucose tolerance or β-cell function in type 2 diabetes [129].

Other reasons for the easy induction of oxidative stress in β-cells may come from the specific details of FA metabolism, if a particular reaction is inhibited or overloaded. Thus, for example, the pancreatic β-cells contain only two out of five isoforms of long-chain acyl-CoA synthetases (ACSLs), ACSL4 and ACSL3, which convert FAs to fatty acyl-CoAs. Their silencing deteriorated GSIS by ~30% [130], evidencing that a slow conversion of non-esterified LCFAs into acyl-CoAs can already set cytosolic-free LCFA concentrations close to the threshold, when the insulin secretion is blocked. Simply, the resulting LCFA accumulation without their further metabolism was the inhibitory factor.

One reason might be that the ACSL capacity in the intact β-cells is already saturated and thus, after overcoming a certain threshold, the LCFAs accumulate as an ACSL substrate. Hypothetically, this might lead to the inhibition of GSIS, but also of FASIS. The glycerolipid/FA cycle might be inhibited, thus inhibiting one leg of the mechanism for insulin granule exocytosis. Another leg of FASIS (i.e., β-oxidation), is set at the level below the threshold and the additional β-oxidation is blocked when the free LCFAs are not converted to acyl-CoAs, and then to acyl-carnitines, and enter the mitochondrial matrix. Moreover, apparently, the remaining GPR40 signaling does not profit from the ongoing LCFA accumulation. However, the mechanism of inhibition is still speculative. Thus, the apoptosis-free threshold for lipotoxicity is yet to be determined and the relevant mechanisms have to be revealed. It has also been found that FA β-oxidation is suppressed by FoxO1 deacetylation by certain sirtuins, while GSIS was sustained [131].

Concerning the mitochondrial mechanism of LCFA lipotoxicity, specifically with regard to GSIS, excessive uncoupling because of the extensive stimulation of mitochondrial uncoupling proteins, including UCP2, may reduce OXPHOS and hence ATP synthesis, and thus prevent the correct functioning of the glucose sensor of pancreatic β-cells [2]. At the excessive free FA overload in pancreatic β-cells, other carriers of the mitochondrial anion carrier SLC25 gene family, such as the ADP/ATP carrier (adenine nucleotide translocase), can also mediate FA-induced uncoupling [132,133,134]. Moreover, the FA overload can contribute to the induction of apoptosis that is related to the so-called permeability transition opening, just via the ADP/ATP carrier participation. These effects are also correlated with the dysfunctional Ca^2+^ homeostasis, specifically in mitochondria [135,136].

With the MIN6 insulinoma cell line, treatment has been demonstrated by a FA mixture that mimics the analytical composition of the metabolic syndrome; elevated LCFAs reduced insulin secretory function after 27 h, decreased viability, and reduced also mitochondrial energy metabolism and induced fission. The used FA mixture also increased lipid peroxidation and reduced the antioxidant capacity of MIN6 cells [137]. Note that the used FA mixture was taken as a knowledge-base from the previously obtained FA profiles of obese adolescent subjects with metabolic syndrome, and, as such, might reflect existing inflammatory status, insulin resistance, and adrenergic hypersensitivity manifested by hypertrophied adipocytes.

Incubations of INS-1E cells or isolated rat pancreatic islets with high glucose plus palmitic acid led to an exchange with acyl residues of phospholipids, while the released PUFAs were peroxidized [138]. The products of lipid peroxidation, such as 4-hydroxynonenal at lower FA doses, stimulated PPARδ and augmented the insulin secretion. However, high palmitic acid dose at high levels of glucose induced ER stress, which overcame the activating effect and profoundly suppressed insulin secretion.

#### 3.1.4. Lipotoxicity Due to a Type of FA Species

Not only the local FA concentrations or amounts bound to albumin or contained in chylomicrons and lipoproteins, but also the type of FA species, matter, in some cases for lipotoxicity. In model RINm5F and INS-1E cells, saturated FAs usually exhibit a strong cytotoxic effect, whereas unsaturated FAs and PUFAs seem to be nontoxic [48,49]. For primary pancreatic β-cells, unsaturated FAs seem to be equally toxic [48,49]. In the following chapters, we list some examples as well as findings that PUFAs, under certain circumstances, may prevent the toxicity of, for example, saturated FAs.

In addition to mitochondria, peroxisomes have emerged as key regulators in overall cellular lipid and ROS metabolism [139,140]. Besides the crosstalk between mitochondrial and peroxisomal β-oxidation, both of which are enzymatically equipped for degradation of saturated and unsaturated FAs [141], the oxidative stress that is preferentially induced in peroxisomes due to the FA metabolism was also considered to apparently distinguish between saturated and unsaturated FAs [48,49]. It has been reported that peroxisomes increase H_2_O_2_ formation responding to FAs [124,141], but they contain distinct enzymes for each particular step of β-oxidation and have different substrate specificities compared to mitochondria [142]. For example, dietary FAs such as palmitic acid, oleic acid, and linoleic acid are preferentially metabolized in mitochondria, whereas very-long chain fatty acids, which cannot be oxidized in mitochondria, are substrates for peroxisomal β-oxidation [139,143]. Despite these metabolic preferences, the work of Lenzen’s group indicates that the metabolism of long-chain saturated nonesterified fatty acids leads to H_2_O_2_ formation in peroxisomes rather than in the mitochondria, and proposes a new concept of fatty acid-induced β-cell lipotoxicity that is mediated via ROS formation through peroxisomal β-oxidation [73,144,145,146]. Currently, the importance of cooperation between the mitochondria and peroxisomes is becoming widely recognized; however, the molecular mechanisms that are underlying the metabolic and redox interplay between the mitochondria and peroxisomes are still poorly understood (recently reviewed by Lismont et al. [147]).

Additionally, the length of the LCFA is at stake. For example, an endoplasmic reticulum enzyme Elovl6, which converts C16 saturated and mono-unsaturated FAs into C18 species, has recently been recognized as another fundamental factor linking dysregulated lipid metabolism to β-cell dysfunction, islet inflammation, and β-cell apoptosis [148]. These findings highlight oleate as exhibiting a higher lipotoxic efficiency in the etiology of type 2 diabetes. Recognition that longer LCFAs are ‘safer’ has also been supported by the finding of silencing of elongase of very long chain fatty acids 2, Elovl2, diminished GSIS [149].

In contrast, EPA and DHA increased the intracellular insulin content and reportedly reduced superoxide production (monitored by the dihydroethidine staining) and prevented the NO increase that was induced by palmitic acid in INS-1E cells. These effects were explained by the EPA- and DHA-stimulated expression of antioxidant enzymes such as GPX1 [150].

#### 3.1.5. Lipotoxicity Due to Lower Antioxidant Enzyme Expression and Function

Pancreatic β-cells are known to contain low levels of antioxidant enzymes and redox buffers. This natural setting allows fine redox regulations on the one hand, but, on the other hand, this makes β-cells exceptionally vulnerable to a further reduction in antioxidative enzymes or redox buffers. The expression of cytoprotective genes [151,152] and proteins and/or activity of antioxidant enzymes is low in rodent β-cells compared with other organs (see [128]). When compared to liver content, pancreatic islets contain only 1% catalase, 2% GPX, and 29% CuZnSOD activities [81,151,152,153]). SODs might be considered as prooxidative, namely, when playing a role in redox signaling. SODs are prooxidative when the downstream degradation of H_2_O_2_ is insufficient [48]. MnSOD, being locally highly concentrated in a small mitochondrial matrix compartment, thus locally forms H_2_O_2_. 

Additionally, a rather low repair machinery for oxidatively damaged DNA is specific for β-cells [154]. In contrast, β-cells are rich in peroxidase-based antioxidant defenses, such as glutaredoxin and thioredoxin [155]. Human β-cells are less prone to oxidative stress, because they possess greater catalase and SOD activities [156]. Yet, GPX activity is rather low in human islets [157]. Nevertheless, glutathione provides an important mechanism that protects the β-cells against oxidative damage, in addition to vitamin E, ascorbate, and uric acid, among small antioxidant molecules [158].

Mitochondrial MnSOD (SOD2) is physiologically inactivated by acetylation, while NAD^+^- dependent deacetylases in the mitochondrial matrix, such as sirtuin-3, restore its activity [159]. Indeed, overacetylated MnSOD has been found after the palmitate treatment of INS-1E cells and human pancreatic islets [160]. In connection with the pro-oxidant effect of a high-fat diet, it has been found that sirtuin-3 expression is reduced in β-cells after a high-fat diet. In accordance with this, GSIS was reduced and increased the H_2_O_2_ aggravated c-Jun N-terminal kinase, whereas both were rescued by the sirtuin-3 overexpression [161]. It has to be noted that the pro-OXPHOS function of all of the mitochondrial (and in certain cases cytoplasmic) sirtuins should always improve GSIS, since the glucose sensor is indeed dependent on OXPHOS. Thus, this is the mechanism by which sirtuin-3 attenuates the palmitate-induced lipotoxicity [162].

Interestingly, being localized at peri-plasma membrane cytosol, glutaredoxin GRX1 has also been implicated in the modulation of Ca^2+^-dependent insulin exocytosis, which was suppressed by GRX1 silencing [83]. The stimulatory action of NADPH on the exocytotic machinery was found to correlate with ~30% inhibition in whole-cell Ca^2+^ currents. Upon GRX1 silencing, NADPH did not amplify the insulin release, but still inhibited Ca^2+^ currents.

Concerning the ER antioxidant protection, the ER-resident GPX7 and GPX8 are not expressed in rat β-cells. This allows higher intensity of, for example, a palmitate induction of ER stress and apoptosis of β-cells [163]. It is also possible that the composition of FAs in obese people with metabolic syndrome can induce changes in the fluidity of microsomal membranes, impairing ER function and thus also insulin secretion.

#### 3.1.6. Chronic LCFA Lipotoxicity

LCFAs alter cell signaling [164] and membrane composition in β-cells, and these and other phenomena substantiate the chronic lipotoxicity. The chronic lipotoxicity of LCFAs related to β-cells may arise also from the overstimulation of their GPR receptors and from the lasting oxidative stress that is provided by more frequent FA β-oxidation. Chronic lipotoxicity leads to dysbalances in the β-cell housekeeping, autocrine or paracrine hormonal responses, and β-cell biogenesis. Additionally, the ER calcium depletion that is induced by saturated FAs and cytokines causes β-cell ER stress and apoptosis. Indeed, oxidative stress and ER stress have been traditionally considered to play a crucial role in gluco/lipotoxicity. Excessive LCFAs also stimulate numerous transcriptional changes via the interaction with the nuclear orphan Nur 77, which possesses a repressive role in insulin gene regulation, thus providing another important line leading to lipotoxicity, besides cellular stress and inflammation consequences [165]. Other studies have shown the activation of stress kinases, such as JNK, IKK, PKR, PKC, and others, inducing intracellular pro-inflammatory pathways. Likewise, the NF-κB-dependent inflammatory signals are strongly affected by the increased ROS levels that may be related to the FA overload. Thus, palmitate causes wide changes in the gene transcription via NF-κB signaling [166].

Chronic lipotoxicity is exerted typically by saturated FAs, whereas PUFAs exert rather antilipotoxic effects [167]. Thus, a long-term treatment of β-cell with arachidonic acid increases GSIS and β-cell survival [168]. In contrast, chronic palmitate treatment inhibits GSIS and impairs insulin gene expression by decreasing the activity of its promoter or diminishing the binding of pancreas duodenum homeobox-1 and MafA to the preproinsulin gene-flanking sequence [169]. To simulate experimental chronic lipotoxicity, isolated human pancreatic islets were exposed for a week to elevated palmitate. The GSIS was reduced after a week, and the insulin content decreased (the proinsulin to insulin ratio doubled). The proteins that were involved in lipid and/or cholesterol biosynthesis upregulated their expression, but the proteins of immature secretory granules decreased [170]. The early stage of lipotoxicity in mice was evoked by dietary palmitic acid-supplementation for two weeks, and this treatment reduced the GSIS and induced ER stress in pancreatic islets [171].

Chronic palmitate treatment also caused the dissociation of Ca^2+^ channels from secretory granules, which impairs IL–he insulin exocytosis [172]. Palmitate impairs and reduces the PDX-1 and GLP-1 receptor expression and signaling in β-cells via an increased expression of a sterol regulatory element-binding protein, SREBP-1C [173]. Palmitate-induced apoptosis was found to be prevented by the activation of the PPARβ/δ receptors via the upregulation of the GLP-1 receptor [174]. The prevention of the unfolded protein response (i.e., ER stress) was also protective [175]. Finally, long-term palmitate treatment has been found to affect cAMP signaling in pancreatic β-cells [176].

Stearic acid seems to be even more lipotoxic, since, via the stimulation of miR-34a-5p, it represses BCL2 and BCLw antiapoptotic proteins. This mechanism involves p53 and the activation of protein kinase-like endoplasmic reticulum kinase (PERK) by stearic acid [177].

In insulinoma INS-1E cells, a hydroxy-FA, 20-HETE, was reported to activate the AKT/GSK-3β pathway and to reduce the expression of GLUT2, which diminishes GSIS [178]. Finally, the proliferation that is otherwise stimulated by glucose is inhibited by LCFAs by inducing the cell cycle inhibitors p16 and p18 [179]. Human pancreatic islets also provide connectivity among all of the β-cells, strengthened by incretins, which is, however, disrupted by lipotoxicity [180]. Concerning human studies, one has to note that high body mass index subjects typically possess a higher mass of β-cells. Thus, fasting serum LCFA levels are correlated with long-term progressive deterioration of insulin secretion, as reported for Japanese patients with type 2 diabetes [181].

### 3.2. Chronic Low-Grade Inflammation Related to LCFA Metabolism in β-Cells

In this review, we focus on pancreatic β-cells, hence the systemic low-grade inflammation is only briefly discussed in Section 3.2.1. However, the reader can find excellent reviews, exemplified by [6,7,8]. Pancreatic islets are infiltrated with immune cells upon islet inflammation, which has long been related to type 2 diabetes [182]. This is accompanied by the increased expression of cytokines and chemokines by both immune cells and islets cells, including β-cells. Interestingly, FAs induce islet (β-cell) inflammation (Section 3.2.2), which in an exaggerated pro-inflammatory milieu, leads to β-cell impairment and apoptosis, or other forms of cell death, as described in Section 3.2.3. Nevertheless, one has to keep in mind that islet inflammation, specifically infiltration with M2 macrophages, should also exert a resolving function and consequently, the beneficial effects on β-cells.

#### 3.2.1. Systemic Pro-Inflammatory Roles of Fatty Acids

Chronic low-grade systemic inflammation and ectopic lipid accumulation in non-adipose tissues, sometimes referred to as metabolic inflammation or meta-inflammation, have been implicated in the development of insulin resistance [6]. The systemic inflammatory response originates usually in WAT, which produces a variety of inflammatory cytokines and chemokines, called adipokines, such as leptin and adiponectin. This enables the bloodstream transfer of pro-inflammatory signals to other tissues, such as the liver, pancreas, hypothalamus, and skeletal muscle, to establish more complex inflammation, as it exists in type 2 diabetes.

The western diet contains a high amount of ω6-PUFAs, particularly arachidonic acid. Consequently, ω6-PUFAs accumulate in the membrane phospholipids of cells in tissues. However, arachidonic acid is also a precursor of a number of potent pro-inflammatory mediators, including leukotrienes and prostaglandins. These are subsequently responsible for inflammatory responses, such as vasodilation and leukocyte migration. It is believed that increased ω6-PUFAs, either arachidonic acid, or its precursor linoleic acid, may increase inflammation [183]. Nevertheless, studies in healthy human adult subjects did not show this trend and, for example, prostaglandin PGE_2_, was shown to also exert anti-inflammatory effects, which has been documented by the reduced interleukin (IL)-1 and TNFα production.

In contrast, ω3-PUFAs, including linolenic acid and longer-chain PUFAs, EPA, and DHA, being part of cell membranes as well, have all been suggested to counteract inflammation, obesity, and insulin resistance development by modulating mitochondrial bioenergetics and ER stress. Specifically, ω3-PUFAs increase mitochondrial biogenesis through proliferator-activated receptor gamma coactivator (PGC1α) and nuclear respiratory factor 1 (NRF1) [184,185,186]. This regulation also increases the FA β-oxidation in mitochondria through the enhanced expression of palmitoyl transferase CPT1, by AMPK activation. Consequently, this could lead to the decreased ectopic lipid accumulation and systemic lipotoxicity.

Saturated FAs, such as palmitic and stearic acids, counteract the regulations by ω3-PUFAs mainly in skeletal muscle, liver, heart, and visceral fat. Moreover, in epidydimal fat (visceral fat), fat synthesis is suppressed, while there is a significantly increased loss of energy in the form of heat, which is caused by the enhanced UCP3 activity and peroxisomal acyl-coA oxidase expression [187]. Although saturated FAs can induce ER stress and apoptosis, leading to inflammation and degeneration, the supplementation of ω3-PUFAs was shown to be protective and can even ameliorate insulin resistance in high-fat diet-induced obese mice [188]. It was suggested that the ω3-PUFAs-mediated suppression of ER stress in adipocytes is due to the AMPK activation. 

Another mechanism was described for ω3-PUFAs during the suppression of pro-inflammatory action, through their initial binding to the GPR120 receptor in macrophages [189]. It has been demonstrated that the GPR120 receptor activation can induce NLRP3 inflammasome through ER-mitochondria juxtaposition regulation, with the participation of calcium mobilization. Whereas the saturated FAs induce NLRP3 inflammation activation (through generated ceramides), and the monounsaturated and PUFAs exhibit the inhibitory role. Additionally, GPR40 signaling takes place in neutrophils [190].

It should be noted that ω3-PUFAs are essential and need to be externally supplemented to the body. Consequently, their optimum dietary content seems to be a prerequisite for a healthy function of adipose tissue and metabolic peripheral tissues. Thus, lipoprotein lipase is an important regulator of PUFA delivery to the peripheral tissues from triglyceride-rich lipoproteins. Interestingly, the interaction of ω3-PUFAs and ω6-PUFAs and their lipid mediators, in the context of inflammation, is quite complex. It has been reported that a diet rich in ω6-PUFAs can inhibit the anti-inflammatory effect of ω3-PUFAs. These interrelationships need to be further studied.

Interestingly, short chain FAs (below six carbons), which are produced and released by the colonic fermentation processes in the gastrointestinal tract, were shown to be able to reduce the pro-inflammation in human adipose tissue. Propionate, as a major microbial fermentation metabolite, was shown to induce the satiety hormone leptin and to reduce the expression of inflammatory cytokines, namely chemokines, from adipose tissue through GPCR41 and GPCR43 receptors [191].

#### 3.2.2. Fatty Acids and Pancreatic Islet Inflammation

**Figure 4:**
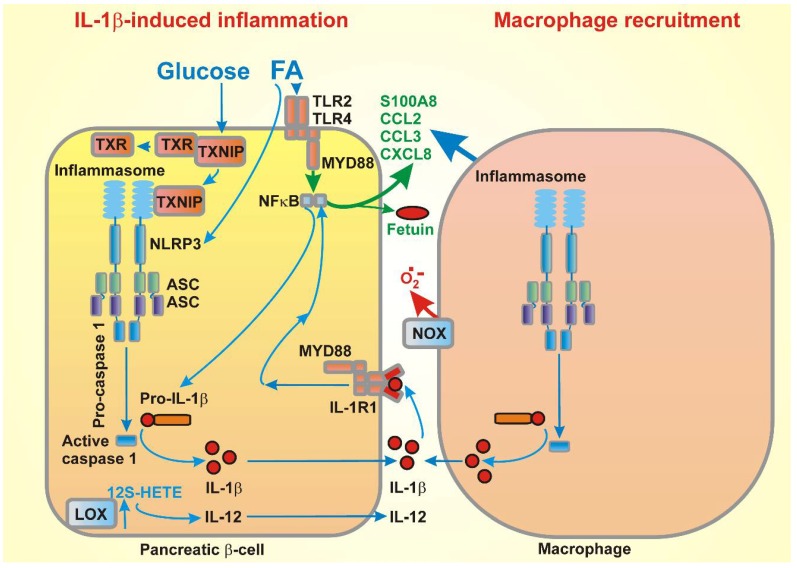
FA-stimulated cytokine release in β-cells, followed by macrophage recruitment, as follows: example of stimulation of IL-1β expression by the concerted effect of glucose and FAs (via thioredoxin-interacting protein (TXNIP) and toll-like receptors (TLR), respectively), including the stimulation of the expression of exemplar chemokines, is illustrated with the consequences for subsequent macrophage recruitment and induction of both the cytokine expression and superoxide release from the M1 macrophages into intersticia. Note that not only the depicted cytokine or chemokine types are secreted. The illustrated examples were chosen for simplicity. For information of the complete pattern, please refer to the text (Section 3.2).

The LCFAs seem to be a master regulator of inflammation in β-cells (Figure 4). Thus, for example, the blockage of the NF-κB/MIF inflammatory pathway by fenofibrate can diminish FA lipotoxicity, including β-cell dysfunction and apoptosis [192]. LCFAs, together with the stimulation of the toll-like receptor 4 (TLR4), promote a plethora of pro-inflammatory factors in β-cells. Additionally, Fetuin-A secretion that is induced by palmitate has been reported to be induced via the NF-κB/MIF inflammatory pathway. Fetuin-A is an α-2-HS-glycoprotein that is typically secreted by the liver. In pancreatic islets, Fetuin-A secretion then establishes ‘an inflammatory environment’, leading to β-cell dysfunction [193].

Additionally, the major lipoxygenase in human pancreatic islets, 12-LO, encoded by the *Alox*12 gene, produces 12-S hydroxyeicosatetraenoic acid (12-S HETE) from arachidonic acid. The increased production of 12-S-HETE in pancreatic islets and adipose tissue has been reported in rodent diabetes models and in diabetic patients. It exerts inflammatory effects by inducing cytokine production, such as IL-12, activating c-Jun kinase and oxidative stress pathways by p38 MAPK-induced NADPH oxidase (NOX1) activity. As a result, the β-cell viability is impaired as well as the insulin secretion [194].

In type 2 diabetes, the number of macrophages increases in the pancreatic islets, thus contributing to the elevation of pro-inflammatory cytokines within the islets [195]. Nevertheless, inflammatory pathologic changes are not found in all of the type 2 diabetic patients [195]. It may be therefore questioned whether proinflammatory cytokines are released and whether macrophages are activated in human pancreatic islets. The difference from rodents may also originate from the fact that a large mass of human β-cells is not associated within islets.

In addition, chemokines are critical for the recruitment of immune cells into pancreatic tissue, and for facilitating the expression and signaling changes in the resident leucocytes [196]. Saturated FAs (e.g., palmitate) induce chemokine and cytokine (CXCL1 and CCL3, plus IL-6 and IL-8) expression within the pancreatic islets in vitro [197,198]. Moreover, the palmitate-induced expression of inflammatory cytokines, IL-8 and IL-6, in human pancreatic islets was IL-1-dependent, since IL-1R abolished the effects of palmitate. Inflammatory cytokines were shown to be produced by macrophages in islets, which then promote β-cell dysfunction and enhance chemokine expression in β-cells. IL-1β has a paracrine or autocrine effect. On the other hand, the activation of pattern recognition receptors, including TLR4 in pancreatic islets by palmitate in β-cells, has been shown to be responsible for the initial chemokine secretion that induces macrophage recruitment [199]. Thus, communication via inflammatory cytokines and chemokines between the M1-like macrophages and β-cells form a vicious cycle that amplifies pancreatic islet inflammation.

Recently, the S100 calcium-binding protein A8 (S100A8), a member of the damage-associated molecular pattern molecules, has been implicated in β-cell inflammation [200]. The TLR4 signaling increased the expression of S100A8 in pancreatic islets upon palmitate stimulation during their co-culturing with unstimulated peritoneal macrophages at high levels of glucose. S100A8 mediated the interaction between the islets and macrophages, and induced β-cell apoptosis, which was decreased by the inhibition of the TLR4 pathway.

Additionally, medical data are available. The upregulation of cytokine and chemokine transcription has been reported for the islet donors with type 2 diabetes [201,202]. Analyses of laser microdissected β-cells of type 2 diabetic donors have shown an upregulation of cytokines IL-1β, IL-8, and IL-11, and chemokines CCL2, CCL11, and CCL13, as well as an IL-1α cytokine downregulation [203]. Notably, IL-1β was not found by the in situ hybridization of the non-diabetic control samples [204].

#### 3.2.3. Consequences of Exaggerated Pro-Inflammatory Milieu

Macrophages represent a spectrum of cells with a functional plasticity that can be classified between the extreme specificities of ‘classically’ activated M1 macrophages, which are cytotoxic and produce pro-inflammatory cytokines; and ‘alternatively’ activated M2 macrophages, resolving inflammation and repairing the tissues by phagocytosis of apoptotic cells and remodeling of the extracellular matrix [205]. The M1/M2 proportion in diabetic pancreatic islets has not yet been satisfactorily evaluated; nevertheless, one may predict that at the M1 macrophage, a more than sufficient deterioration of β-cells may occur.

It has been indicated that the infiltration of the M1 macrophages causes a significant loss of β-cells [206]. For example, endocannabinoids, through the activation of the NLRP3 inflammasome in infiltrating macrophages, contribute to the decreasing β-cell number in type 2 diabetes [207]. Typically, M1 macrophages, upon classical activation, produce inflammation-dependent ROS and add to the already existing oxidative cells within the affected β-cells, with consequences as described in Section 3.1. This is, however, manifested typically for type 1 diabetes. Thus, the pro-inflammatory cytokines, such as IL-1β, clearly induce apoptosis of pancreatic islet β-cells, revealed as cytochrome *c* release from mitochondria followed by the activation of downstream caspases [198]. Elevated ROSs are mediators of cytokine-induced cell death, since the overexpression of antioxidant enzymes prevented the β-cells from cytokine-induced death [208]. Cytokines also induce ER stress by several mechanisms [209].

### 3.3. Native Antilipotoxic Factors

Finally, phylogenesis has developed factors counteracting lipotoxicity, with a mission to protect pancreatic β-cells. Let us briefly describe several of them in the following sub-sections.

#### 3.3.1. Incretins

GLP-1 prevents β-cell death by increasing autophagic flux, which improves lysosomal function that would be otherwise impaired by lipotoxic and glutotoxic stimuli. These stimuli lead to the accumulation of defective lysosomes and cathepsin D release, which contributes to cell death [210]. The beneficial effects of incretins have been described elsewhere [13,14].

#### 3.3.2. Irisin

Recently, myokine irisin has been recognized as another pancreatic β-cell secretagogue and as a survival factor [211]. Irisin potentiates GSIS via the PKA pathway. As a pro-survival factor, irisin counteracts the LCFA-induced β-cell apoptosis via AKt/Bcl2 signaling, and increases β-cell proliferation.

#### 3.3.3. Neutral Ceramidase

Neutral ceramidase-degradating ceramides are suppressed by saturated FAs; thus, when there is an excess of saturated FAs, ceramides are accumulated in β-cells [212]. This leads to the facilitation of apoptosis that is promoted by saturated FAs. In conclusion, sufficient neutral ceramidase activity is required to defend lipotoxicity.

#### 3.3.4. Other Native Antilipotoxic Factors

The ER-localized protein thrombospondin 1 (THBS1) has also been identified as a pro-survival factor upon lipotoxic stress of β-cells. THBS1 has been found to be cytoprotective to rat, mouse, and human β-cells during cytokine- or thapsigargin-induced ER stress. The mechanism involves the expression maintenance of the mesencephalic astrocyte-derived neutrotrophic factor (MANF) in β-cells. MANF prevents the pro-apoptotic BH3-only protein BIM from triggering apoptosis [213].
